# Bluetongue virus non-structural protein 1 is a positive regulator of viral protein synthesis

**DOI:** 10.1186/1743-422X-9-178

**Published:** 2012-08-29

**Authors:** Mark Boyce, Cristina C P Celma, Polly Roy

**Affiliations:** 1 Faculty of Infectious and Tropical Diseases, London School of Hygiene and Tropical Medicine, Keppel Street, London, WC1E 7HT, UK; 2 Present Address: The Institute for Animal Health, Pirbright Laboratory, Ash Road, Pirbright Surrey GU24 0NF, UK

**Keywords:** Bluetongue, NS1, Translation, Protein synthesis

## Abstract

**Background:**

Bluetongue virus (BTV) is a double-stranded RNA (dsRNA) virus of the *Reoviridae* family, which encodes its genes in ten linear dsRNA segments. BTV mRNAs are synthesised by the viral RNA-dependent RNA polymerase (RdRp) as exact plus sense copies of the genome segments. Infection of mammalian cells with BTV rapidly replaces cellular protein synthesis with viral protein synthesis, but the regulation of viral gene expression in the *Orbivirus* genus has not been investigated.

**Results:**

Using an mRNA reporter system based on genome segment 10 of BTV fused with GFP we identify the protein characteristic of this genus, non-structural protein 1 (NS1) as sufficient to upregulate translation. The wider applicability of this phenomenon among the viral genes is demonstrated using the untranslated regions (UTRs) of BTV genome segments flanking the quantifiable Renilla luciferase ORF in chimeric mRNAs. The UTRs of viral mRNAs are shown to be determinants of the amount of protein synthesised, with the pre-expression of NS1 increasing the quantity in each case. The increased expression induced by pre-expression of NS1 is confirmed in virus infected cells by generating a replicating virus which expresses the reporter fused with genome segment 10, using reverse genetics. Moreover, NS1-mediated upregulation of expression is restricted to mRNAs which lack the cellular 3^′^ poly(A) sequence identifying the 3^′^ end as a necessary determinant in specifically increasing the translation of viral mRNA in the presence of cellular mRNA.

**Conclusions:**

NS1 is identified as a positive regulator of viral protein synthesis. We propose a model of translational regulation where NS1 upregulates the synthesis of viral proteins, including itself, and creates a positive feedback loop of NS1 expression, which rapidly increases the expression of all the viral proteins. The efficient translation of viral reporter mRNAs among cellular mRNAs can account for the observed replacement of cellular protein synthesis with viral protein synthesis during infection.

## Background

Bluetongue virus (BTV) is the prototype member of the *Orbivirus* genus of the *Reoviridae* family. BTV is an arbovirus vectored between ruminant hosts by biting midges of the *Culicoides* genus [1,2], causing disease in wild and domesticated ruminants, including sheep, goats and cattle. The virus has a global distribution which has extended into mainland Europe since 1998 and has caused a severe economic impact on European livestock farming [3,4]. BTV has a segmented genome consisting of ten linear double-stranded RNA (dsRNA) molecules [5,6]. Like other members of the *Reoviridae* the viral messenger RNA (mRNA) is synthesised by transcription of the double-stranded RNA (dsRNA) genome segments which remain packaged within the uncoated core particle [7-9]. The ten viral mRNAs are synthesised as complete end-to-end copies of the genome segments by the RNA-dependent RNA polymerase (RdRp) component of the transcription complexes, VP1, present at the vertices of the core particle [9-11]. Before extrusion into the cytosol each transcript is capped and methylated by VP4 at the 5^′^ end, to produce a cap1 structure (^7^mGpppGm) identical to that found in cellular messenger RNAs [12-14]. The 5^′^ cap1 structure is almost ubiquitous among cellular mRNAs and is important for mRNA stability and efficient translation [15]. The 3^′^ end of viral transcripts lack a poly(A) tail, which normally functions in eukaryotic mRNAs to increase the rate of translation initiation and RNA stability [15-18]. Following the infection of mammalian cells BTV proteins rapidly accumulate to be the most abundant cytoplasmic proteins, with the viral proteins being the major products of active translation by 8 hours post-infection [19]. This rapid replacement of cellular protein synthesis with viral protein synthesis indicates that BTV has effective mechanism(s) for ensuring the translation of its mRNA, but how the viral mRNAs of orbiviruses effectively compete with cellular mRNAs for translation has not been addressed to date.

A characteristic of *Orbivirus* infections is the accumulation of highly abundant cytoplasmic tubular structures composed of the 64 kDa non-structural protein 1 (NS1), for which no homologue has been found in other genera of the *Reoviridae*[20-22]. NS1 is the most highly expressed BTV protein, becoming the most abundant cytoplasmic protein before completion of the replication cycle. Understanding of the functions of NS1 remains limited to a proposed role in regulating the mechanism of virus release from the mammalian and insect host cells [23]. In this model the high ratio of NS1 to NS3 in mammalian cells is correlated with cell lysis, and the higher level of NS3 expression observed in *Culicoides* cells is correlated with non-lytic release and persistent infection in culture.

Here we use a synthetic mRNA reporter assay system to screen the BTV gene products for upregulation of the expression of a reporter gene consisting of a fusion between the termini of segment 10 and GFP. The mRNA reporter contains GFP flanked by the 5^′^ end (152nt) and 3^′^ end (149nt) of segment 10, creating a reporter mRNA which has both termini of segment 10. Using this approach NS1 is identified as sufficient to upregulate the reporter protein abundance. The specificity of NS1 for viral mRNAs is investigated using reporter mRNAs consisting of BTV untranlsated regions (UTRs) flanking the reporter ORF, and using mRNAs with 3^′^ poly(A) extensions. Using the reverse genetics system we demonstrate that NS1-dependent upregulation of gene expression also occurs in the context of viral replication in infected BSR cells. To our knowledge this is the first report addressing how orbiviruses upregulate their gene expression.

## Results

### The expression of BTV genes increases the expression of a chimeric genome segment 10 - GFP reporter transcript

**Figure 1:**
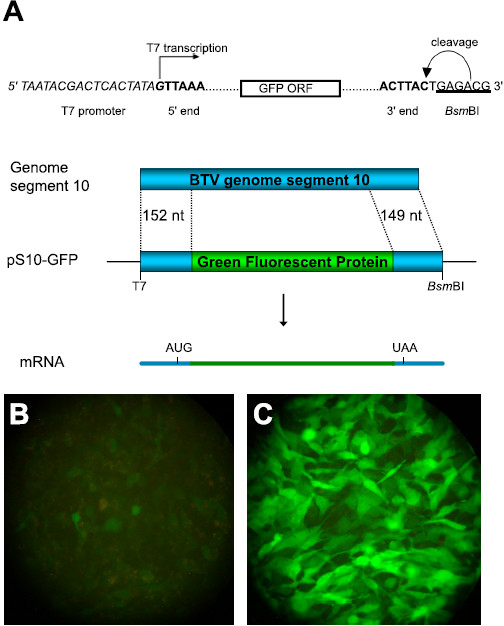
**Expression of BTV genes upregulates expression from capped S10-GFP RNA.** (**A**) pS10-GFP contains the 5^′^ 152nt and 3^′^ 149nt of BTV-10 segment 10 fused in frame with GFP. The 5^′^ end of the capped S10-GFP reporter RNA is defined by the T7 promoter (italicised) and the 3^′^ end is determined by a *Bsm*BI site (underlined). (**B**) and (**C**) Confluent 24 well BSR monolayers fixed in 4% PFA at 22 h post-transfection. (**B**) Transfected with 1 μg capped S10-GFP reporter RNA alone. (**C**) Transfected with 1 μg capped S10-GFP reporter RNA + 0.8 μg BTV-1 mRNA produced *in vitro* from transcribing cores as previously described [35].

It remains unknown how orbiviruses regulate translation of their mRNAs, and which viral proteins are involved. To investigate the regulation of gene expression an RNA reporter assay was designed using a capped reporter transcript, S10-GFP, containing the GFP ORF fused between the 5^′^ and 3^′^ ends of genome segment 10 (Figure 1A). The BTV 5^′^ end sequence was generated using a T7 promoter, and the BTV 3^′^ end sequence was generated using a *Bsm*BI cleavage site as previously described in the establishment of a BTV reverse genetics system [24]. To express all the BTV genes without the rapid lysis produced by BTV infection, viral mRNA generated *in vitro* by transcribing cores were used. Confluent BSR monolayers were transfected with capped S10-GFP reporter RNA alone or in combination with viral mRNA to provide the complete complement of BTV-1 genes. In the absence of BTV gene expression the S10-GFP reporter produced a detectable low level of fluorescence, but coexpression of the BTV gene products increased the level of GFP markedly (Figure 1B and C). The increased accumulation of the reporter strongly suggests that the BTV genome encodes a factor which increases the expression of the NS3 protein encoded by segment 10.

### NS1 expression is sufficient to increase the expression of the segment 10 - GFP reporter transcript

**Figure 2:**
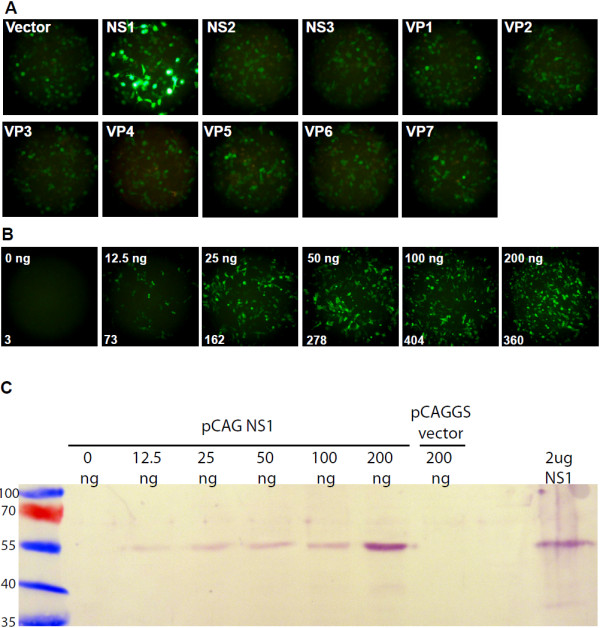
**Expression from capped S10-GFP RNA is upregulated by NS1.** (**A**) BSR 96 well monolayers transfected with 100 ng BTV pCAG clones or empty pCAGGS vector together with 100 ng capped S10-GFP reporter RNA. Fluorescence was recorded at 20 h after transfection with capped S10-GFP reporter RNA. (**B**) Dose–response relationship of expression from capped S10-GFP RNA across a two-fold dilution series of NS1 expression. BSR 96 well monolayers transfected with 0 ng - 200 ng pCAG NS1 and the capped S10-GFP reporter RNA. Fluorescence was recorded at 20 h after transfection with S10-GFP reporter RNA at low magnification (10x objective lens) to maximise the number of cells within the field of view. Values indicate the number of fluorescing cells within the field. (**C**) Expression of NS1 in BSR 96 well monolayers transfected with the same quantities of pCAG NS1 used in panel B. Transfected monolayers were harvested at 18 h after transfection and analysed by SDS-PAGE, followed by immunoblotting using anti-NS1 rabbit polyclonal antibody R633. The quantities above the lanes indicate the amount of plasmid transfected. NS1 positive control -2 μg BTV-10 NS1 purified from Sf9 cells infected with recombinant baculovirus Ac BTV NS1. The numbering on the left indicates the molecular weight of the size markers.

Each BTV gene was tested individually to remove the possibility that the increased reporter expression observed (Figure 1) was due to replication of the reporter RNA. To determine whether any BTV gene product individually upregulates expression from the S10-GFP reporter RNA, each of the ten BTV-10 genes was expressed from the strong CMV/chicken actin hybrid promoter of the pCAGGS vector [25]. To screen the BTV genes, BSR monolayers were transfected with each pCAG clone 18 hours prior to transfection with the S10-GFP reporter transcript. The fluorescence produced with each BTV protein was recorded 20 hours after transfection with the S10-GFP RNA. Only NS1 expression was found to increase the expression from the S10-GFP reporter RNA significantly and no effect on the expression level was observed with the other nine BTV gene products (Figure 2A). To determine the optimal dose for further study the dose–response relationship was tested across a range of pCAG NS1 quantities (Figure 2B, C). The increased translation of the reporter RNA was detectable at 12.5 ng pCAG NS1 per well and found to increase up to 100 ng (Figure 2B). Significant toxicity was observed with 200 ng pCAG NS1 and a standard quantity of 100 ng at 96 well plate scale was chosen for further investigation.

### NS1 regulated expression is not mediated through interaction with NS3

**Figure 3:**
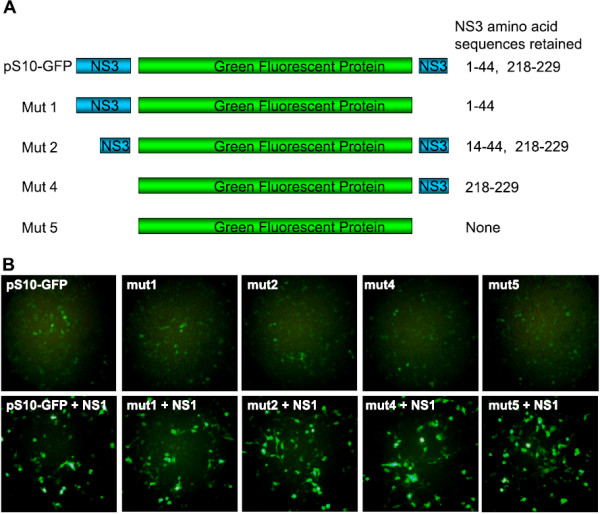
**NS1 does not act through NS3 sequences in the reporter protein.** (**A**) pS10-GFP mutants contain point mutations which reduce or eliminate the regions of NS3 fused to GFP. Mut1, introduced termination codon and −1 frame shift at C-terminus of GFP. Mut2, NS3 initiation codon destroyed. Mut 4, NS3 initiation codon and 2^nd^ methionine codon destroyed. Mut5, NS3 initiation codon and 2^nd^ methionine codon destroyed, and at the C-terminus of GFP a termination codon and −1 frame shift were introduced. (**B**) BSR 96 well monolayers transfected with 100 ng pCAG NS1 and capped S10-GFP RNA mutants. Fluorescence was recorded at 20 h after transfection with reporter RNAs.

The protein encoded by the S10-GFP reporter RNA consists of GFP fused in frame with both termini of the NS3 protein encoded by segment 10. A proposed role for NS1 is its involvement in determining whether lysis is the ultimate fate of the infected cell [23]. BTV infection of mammalian cells is lytic, but in cells derived from the *Culicoides* insect vector the infection is persistent with continual virus release. The ratio of NS3 to NS1 was suggested to control whether lysis occurred, with the higher level of NS3 expression in insect cells preventing lysis induced by NS1 [23]. To address the possibility that the upregulation of the S10-GFP reporter RNA is indicative of an interaction between NS1 and the remaining NS3 protein sequences in the reporter protein a series of S10-GFP variants were made. Each variant retains both the 5^′^ and 3^′^ ends of segment 10, but the length of the NS3 protein sequence fused to the GFP reporter is reduced or eliminated by the destruction of the NS3 initiation codons, or the creation of termination codons or frameshifts (Figure 3A). The expression of all variants was found to be responsive to NS1 including mutant 5 which has no NS3 sequence in-frame with the GFP protein. This demonstrates that NS1 does not increase the abundance of the reporter protein through an interaction with NS3 sequences (Figure 3B), and suggests it acts through the reporter RNA itself.

**Figure 4:**
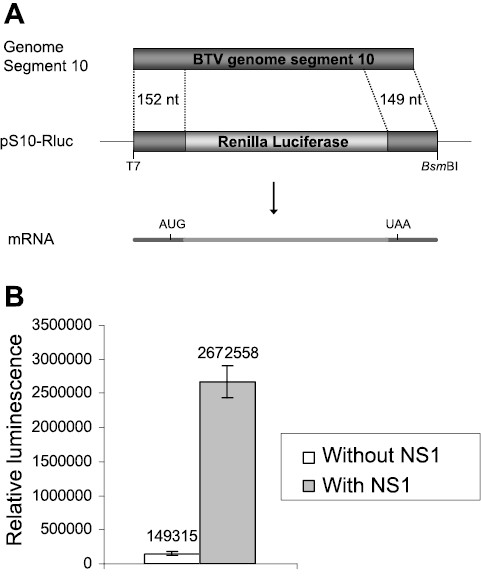
**Expression from capped S10-Rluc RNA is upregulated by NS1.** (**A**) pS10-Rluc contains the 5^′^ 152nt and 3^′^ 149nt of BTV-10 segment 10 fused with the Renilla luciferase gene. (**B**) BSR 96 well monolayers transfected with 100 ng pCAG NS1 and capped S10-Rluc RNA mutants. The amount of luciferase present in the transfected cell lysates was assayed in triplicate at 20 h after transfection with S10-Rluc reporter RNA. Bars indicate one standard deviation from the mean.

To confirm that the choice of reporter gene was not responsible for the observed NS1 dependant increase in abundance, and to generate a sensitive assay with which the level of activation could be quantified, the luciferase based reporter, pS10-Rluc was constructed. The GFP ORF in pS10-GFP was replaced with the Renilla luciferase ORF (Figure 4A). To measure the responsiveness of the S10-Rluc RNA to NS1 the transfections were performed as described for S10-GFP, and the luciferase activity in the cell lysates was quantified. The luciferase expression in S10-Rluc RNA transfected cell lysates was also found to be increased by coexpression of NS1, with a 15–20 fold increase in expression of luciferase being typical of NS1 pre-expression (Figure 4B). The sensitivity gained by measuring luminescence allowed even the relatively low expression of the reporter in the absence of NS1 to be detected at over 10^5^ relative light units, extending the utility of the RNA reporter assay to poorly expressed variants.

### NS1 increases protein expression from reporter mRNAs containing the UTRs of BTV genes

**Figure 5:**
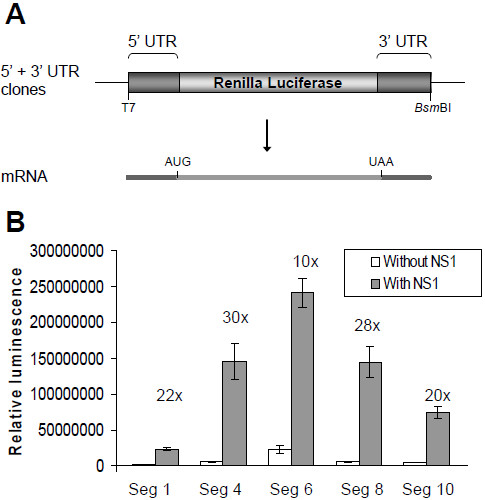
**Expression from capped transcripts containing both UTRs of segments 1, 4, 6, 8 or 10 is responsive to NS1.** (**A**) The 5^′^ + 3^′^ UTR constructs contain the 5^′^ UTR and 3^′^ UTR of BTV-1 segments 1 (VP1), 4 (VP4), 6 (NS1), 8 (NS2), or 10 (NS3) immediately flanking the Renilla luciferase ORF. The 5^′^ end of the capped reporter RNAs are defined by a T7 promoter and the 3^′^ end is determined by a *Bsm*BI site, as described in Figure 1. (**B**) BSR 96 well monolayers transfected with 100 ng pCAG NS1 and capped reporter transcripts. The amount of luciferase was assayed in triplicate 20 h after transfection with the reporter RNAs. Bars indicate one standard deviation from the mean. Fold of NS1-dependent upregulation indicated.

To establish whether NS1 is a specific regulator of expression from segment 10 or has a more general role in viral gene expression a series of reporters were made containing the 5^′^ UTR and 3^′^ UTR of five BTV segments, S1 (VP1), S4 (VP4), S6 (NS1), S8 (NS2) or S10 (NS3) from BTV-1, where the viral ORF is replaced by the Renilla luciferase ORF (Figure 5A). The effect of the UTRs from these segments on the expression of luciferase was measured in the presence and absence of NS1 in the RNA reporter assay (Figure 5B). Each reporter RNA was responsive to NS1, demonstrating that the UTRs are sufficient to confer NS1-responsiveness to the corresponding viral genes, and indicating a general role for NS1 in upregulating the expression of BTV protein. There was variation between the genome segments in the quantity of luciferase expressed from the UTR reporter transcripts in the absence of NS1. This shows that the UTRs of the BTV genes are determinants of the relative expression levels of the viral mRNAs.

### NS1 increases the expression of Renilla luciferase incorporated into the genome of BTV-1

**Figure 6:**
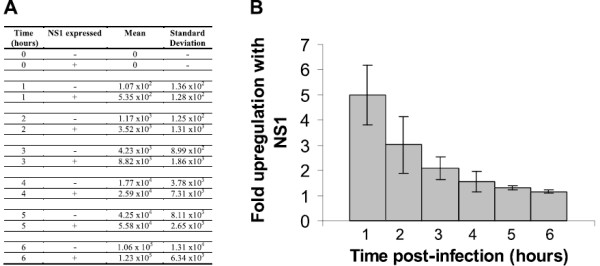
**NS1 upregulates expression of the rescued S10-Rluc reporter segment in replicating BTV-1 S10Rluc.** (**A**) 96 well monolayers were transfected with 100 ng pCAG NS1 or pCAGGS vector, and infected with BTV-1 S10Rluc. The amount of luciferase was assayed in triplicate at 1 h intervals following infection with the BTV-1 S10Rluc reporter virus. Values are relative light units (RLU) expressed as the mean of three replicates. (**B**) Graph of the fold increase of the virus expressed reporter in NS1 expressing cells compared to vector transfected BSR. Values are the fold increase observed when NS1 was pre-expressed. Bars indicate one standard deviation from the mean.

To determine whether NS1 increases the expression of a protein encoded by the BTV genome the chimeric S10-Rluc RNA was recovered into the genome of BTV-1. The S10-Rluc RNA was rescued in the genome of BTV-1 to make BTV-1 S10Rluc virus by using the reverse genetics system with the BSR NS3 line, which complements the absence of functional NS3 [24,26]. The T7 transcripts used in the RNA transfection reporter assay in previous experiments were capped with the anti-reverse cap analogue which is known to substitute for a cap 1 structure in the translation of cellular mRNAs [27]. To determine whether NS1 upregulates expression from an mRNA transcribed and capped by the core particle in the context of virus replication the expression of the Rluc reporter from the viral genome was quantified in infected cells. BSR cells were transfected with pCAG NS1 or pCAGGS and infected with BTV-1 S10Rluc after 20 hours. The amount of luciferase was measured at 1 hour intervals, to quantify the effect of NS1 on expression at early times. In this system also the luciferase activity was found to be higher when BSR cells had been transfected in advance with pCAG NS1, with a marked increase in luciferase expression directly after infection (Figure 6A, B). These data confirmed that NS1 increases expression from transcripts produced from transcribing cores, and showed that the early expression of NS1 is important for the upregulation of viral gene expression.

### 3^′^ poly(A) tailing of mRNA prevents NS1 mediated upregulation

**Figure 7:**
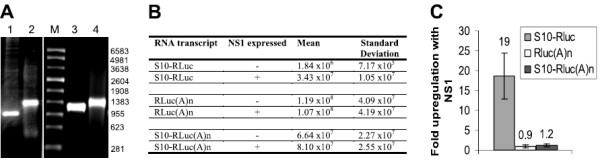
**3**^′^**Polyadenylation of reporter mRNA abrogates NS1-responsiveness.** (**A**) Denaturing 1% agarose gel electrophoresis of T7 transcripts generated from *Xba*I digested pRL-null (Promega) and *Bsm*BI digested pS10-RLuc. Lane 1, Rluc transcript, lane 2, Rluc transcript after 3^′^ polyadenylation with poly(A) polymerase, lane 3, S10-Rluc transcript, lane 4, S10-Rluc transcript after 3^′^ polyadenylation with poly(A) polymerase. M, 1 μg of ssRNA markers (Promega), with length indicated in nucleotides. (**B**) BSR 96 well monolayers transfected with 100ng pCAG NS1 and capped reporter transcripts. The amount of luciferase was assayed in triplicate 20 h after transfection with the reporter RNAs. Values are relative light units (RLU) expressed as the mean of three replicates. (**C**) Effect of 3^′^ poly(A) tail on fold upregulation of expression in response to NS1. Bars indicate one standard deviation from the mean.

The 3^′^ poly(A) sequence is a near-universal feature of cellular transcripts which is absent in the mRNA of orbiviruses. BTV infection replaces cellular protein synthesis with viral protein synthesis [19], but how the cellular gene expression is shut off has not been studied. The efficient competition of viral mRNA translation over cellular translation is one possibility. To address the specificity of NS1-mediated upregulation of gene expression the NS1-responsiveness of a 3^′^ polyadenylated Renilla luciferase reporter with no viral sequences present was tested, as a proxy for a cellular mRNA. The Renilla luciferase transcript was synthesised from *Xba*I-digested pRL-null (Promega) and poly(A) tailed to generate Rluc(A)_n_ mRNA. *In vitro* polyadenylation with *E.coli* poly(A) polymerase was confirmed by the slower migration rate using denaturing agarose gel electrophoresis (Figure 7A). The NS1-responsive expression observed for the S10-Rluc mRNA was not seen with the Rluc(A)_n_ mRNA (Figure 7B, C). To determine whether a 3^′^ poly(A) tail is sufficient for NS1 to discriminate between viral and cellular mRNAs the NS1-responsive S10-Rluc mRNA was polyadenylated (Figure 7A). The resulting S10-Rluc(A)_n_ mRNA was found to be unresponsive to NS1 (Figure 7B, C). The almost complete abrogation of the NS1-responsive phenotype by the addition of a poly(A) sequence demonstrates that the 3^′^ ends of BTV transcripts are critical determinants in the mechanism of NS1 regulated expression. These data show that NS1 is not acting as a global upregulator of translation or of protein stability, but suggest that NS1 specifically increases the expression of viral mRNA over cellular transcripts.

## Discussion

The BTV proteins have been shown to be the most highly expressed genes in BHK cells by 8 hours post infection [19]. The approach used here identifies the tubule forming protein characteristic of orbiviruses, NS1, as a positive regulator of the translation of viral mRNAs. The screen of the BTV genes for proteins which regulate the expression from the segment 10-GFP mRNA identified NS1 as sufficient, and no effect on GFP expression was observed with other BTV proteins. This approach demonstrates that NS1 is a positive regulator of BTV gene expression, but the possibility that complexes containing more than one viral protein also regulate BTV gene expression is not excluded.

The increased expression was also found when the reporter mutants retained the 5^′^ and 3^′^ regions of segment 10, but did not encode the amino and carboxy termini of NS3. The NS1-responsiveness of these variants demonstrates that the short NS3 protein sequences fused to GFP cannot be responsible for the increased accumulation of the reporter protein. This was confirmed by the NS1 responsiveness of transcripts which consist of only the UTRs of BTV mRNAs fused to the Renilla luciferase ORF. Together these results suggest that NS1 acts at the level of the mRNA to either increase its stability and therefore abundance, or increase the rate at which it is used as a template in translation.

The fusion of the Renilla luciferase gene with segment 10 in the S10-Rluc mRNA produced a quantitative and more sensitive reporter system for measuring NS1 responsiveness, with NS1 enhancing the expression from the S10-Rluc mRNA in transfected cells by 15–20 fold. The upregulation of expression from reporter mRNAs containing the UTRs of five BTV genes demonstrates that NS1-dependent gene expression is not restricted to the expression of the NS3 protein from segment 10, but is a wider phenomenon affecting the expression of other BTV genes.

Importantly, the type of transcripts upregulated by NS1 does not extend to the Rluc(A)n mRNA which lacks viral sequences and has a typical cellular 3^′^ poly(A) tail, demonstrating that NS1 is not a general upregulator of expression from all mRNAs. The 3^′^ extension of the NS1-responsive S10-Rluc mRNA with a poly(A) sequence was sufficient to prevent increased expression, showing that the nature of the 3^′^ end of the mRNA is crucial to the upregulation function of NS1. Moreover the absence of upregulation of the polyadenylated mRNAs confirms that NS1 does not increase viral gene expression by globally increasing protein stability. Together these data support a model where NS1 is a positive regulator of viral mRNA expression, but does not increase the expression of cellular mRNAs. The competitive advantage this would give to the translation of viral transcripts can account for the replacement of cellular protein synthesis with viral protein synthesis in BTV infected BHK cells [19].

The BTV UTRs themselves were found be one level of regulation of the expression of the viral proteins, with the absolute levels of expression from each of the UTR-containing reporters varying markedly between the segments. This variation occurred both in the presence and absence of NS1 pre-expression, with NS1 increasing the quantity of luciferase produced from each mRNA by 10–30 fold. The BTV UTRs were found to be determinants of the protein expression level as is established for the UTRs of cellular mRNAs [28], and the upregulatory effect of NS1 is an additional level of regulation. The contribution of the UTRs to the expression level is particularly marked for the segment 6 UTRs which flank the NS1 gene itself, the most highly expressed BTV gene during infection. In this case the level of translation seen in the absence of NS1 is already high, and the absolute quantity of luciferase produced when NS1 is upregulating expression is higher than the other UTRs tested.

When rescued into the genome of infectious virus using the reverse genetics system, the expression of the S10-Rluc reporter genome segment was also responsive to NS1. The expression of NS1 using pCAG NS1 increased the expression of the reporter at early time points, demonstrating that the NS1-dependent upregulation occurs in the context of virus replication where the viral mRNA is synthesised by transcriptionally active cores. The diminishing effect observed with time is an expected consequence of the abundant and rapid synthesis of NS1 by the infecting virus, which would be expected to rapidly exceed the initial difference in NS1 abundance between cells transfected with pCAG NS1 and those transfected with the pCAGGS vector. The higher level of reporter expression seen at early times after infection with the BTV-1 S10Rluc virus demonstrates the competitive advantage given to viral gene expression by the presence of NS1 in the infected cells. Indeed the recovery of viruses by reverse genetics with no NS1 initiation codon, or coding changes which abrogate the regulatory function was not possible (data not shown). Knockdown by siRNA of the moderate level of NS1 expression from pCAG NS1, compared to BTV infection, have been insufficient to reduce reporter activity by more than 2 fold (data not shown). This observation is consistent with the amount of NS1 produced by transfection with small quantities of pCAG NS1 plasmid being sufficient to highly upregulate expression from reporter mRNAs (Figure 2B).

NS1 performing the role of a positive regulator of BTV gene expression is consistent with it being the most highly expressed BTV protein encoded by the most abundant transcript produced by transcribing BTV cores [9,19]. We propose a model of translational regulation in which NS1 increases the expression of the BTV genes, including itself, creating a positive feedback loop of NS1 expression which increases the rate of synthesis of all the viral proteins, as has been found for the rotavirus NSP3 protein [29]. The increased translation of viral transcripts in the presence of NS1 suggests that a competitive advantage is given to the translation of viral transcripts over cellular polyadenylated mRNAs, and is consistent with the observed shutdown of cellular gene expression and its simultaneous replacement with viral gene expression in BTV infected cells by 8 hours p.i. reported by Huismans [19]. Altogether the data presented here demonstrate that the effective competition of viral mRNAs with cellular mRNAs, mediated by NS1, is one way that this is achieved. BTV NS1 and rotavirus NSP3 do not share amino acid sequence homology and are not similar size proteins (64 kDa versus 34 kDa respectively), but NS1 clearly performs the translational upregulation function also reported for rotavirus NSP3 [29-32], although the mechanism leading to increased expression in the presence of NS1 remains to be elucidated. The existence of *cis*-acting sequences within the viral UTRs, particularly the 3^′^ UTR, which allow NS1 to discriminate between viral mRNAs and cellular mRNAs, is one prediction of the model which is being investigated.

## Conclusions

This is the first report addressing how orbiviruses replace cellular protein synthesis with viral protein synthesis. NS1 is identified as sufficient to increase protein synthesis from chimeric virus-reporter gene mRNAs in both transfected and infected cells. The UTRs of segments 1, 4, 6, 8, and 10 are sufficient to confer NS1-responsiveness to protein synthesis. The abrogation of NS1 responsiveness by 3^′^ poly(A) extension of the mRNA demonstrates that the 3^′^ end is a critical determinant of NS1 responsiveness. Knowledge of the structure of NS1 and its interaction with viral mRNAs will be important in understanding how NS1 specifically increases viral protein synthesis in orbiviruses.

## Methods

### Cell lines and virus

BSR cells (a BHK-21 subclone) were cultured in Dulbecco’s modified Eagle’s medium (DMEM) supplemented with 5% (v/v) foetal bovine serum (FBS) at 35°C in 5% CO_2_. The NS3 expressing cell line BSR NS3 was cultured in the presence of puromycin (7.5 μg ml^-1^) [26].

BTV-1 stocks were generated by infecting BSR cells at a multiplicity of infection (MOI) of 0.1 and harvesting the medium at 3 days for wildtype BTV-1 or 7 days for BTV-1 S10-Rluc. Viral stocks were stored at 4°C.

### Infection of transfected BSR cells with BTV-1 S10-Rluc reporter virus

Transfected BSR cells were infected at an MOI of 1 at room temperature for 45 minutes. Wells were washed four times in PBS, and incubated in DMEM supplemented with 5% (v/v) FBS at 35°C in 5% CO_2_.

### T7 promoter plasmid clones used for the synthesis of reporter transcripts

The GFP-containing clone pS10-GFP consists of the 5^′^ 152nt and 3^′^ 149nt of BTV-10 segment 10 fused in frame with the GFP ORF. Mutants of pS10-GFP which did not fuse the remaining BTV protein coding sequences to GFP were generated by site directed mutagenesis using the method of Weiner et al. [33], with the primer pairs S10GFP_mut1F and S10GFP_mut1R (mutants 1 and 5), S10GFP_mut2F and S10GFP_mut2R (mutants 2, 4, and 5), S10GFP_mut3F and S10GFP_mut3R (mutants 4 and 5), S10GFP_mut4F and S10GFP_mut4R (mutants 4 and 5).

The Renilla luciferase-containing clone pS10-Rluc consists of the 5^′^ 152nt and 3^′^ 149nt of BTV-10 segment 10 fused in frame with the Rluc ORF.

The Renilla luciferase clones containing the 5^′^ and 3^′^ UTRs of segments 1, 4, 6, 8 or 10 of BTV-1 consist of the Renilla luciferase ORF immediately flanked by the UTRs.

The pRL-null (Promega) vector contains the Renilla luciferase ORF downstream of a T7 promoter.

### BTV protein expression plasmids

BTV protein expression plasmids were generated by cloning the ten BTV-10 ORFs downstream of the chicken β actin promoter of the pCAGGS expression vector reported previously [25], to generate pCAG VP1, pCAG VP2, pCAG VP3, pCAG VP4, pCAG VP5, pCAG VP6, pCAG VP7, pCAG NS1, pCAG NS2 and pCAG NS3.

### Synthesis of reporter RNAs

Synthetic single-stranded RNAs were prepared by run-off *in vitro* transcription from *Bsm*BI digested plasmid clones, using T7 RNA polymerase. Transcripts were prepared with anti-reverse cap analogue (ARCA) from linear plasmid DNA using the mMESSAGE mMACHINE T7 Ultra kit (Ambion) as previously described [24]. Poly(A) tailed transcripts were generated using poly(A) polymerase according to the manufacturer’s instructions.

### Denaturing agarose gel electrophoresis

Transcripts were analysed under denaturing conditions by electrophoresis on 1% agarose in morpholinepropanesulfonic acid (MOPS) electrophoresis buffer in the presence of formaldehyde [34].

### Immunoblot analysis of pCAG NS1 transfected BSR cells

Samples were resolved in 10% SDS-PAGE gels and transferred to nitrocellulose membranes. Standard immunoblotting techniques were used with rabbit polyclonal antiserum R633 raised against NS1. Proteins were visualised using alkaline phosphatase-conjugated goat anti-rabbit IgG (Sigma) with BCIP/NBT substrate (Sigma).

### Purification of BTV core particles

BSR cultures were infected with BTV at an MOI of 0.1. Transcriptionally active BTV-1 core particles were purified as previously described and stored at 4°C [35].

### Synthesis and purification of BTV mRNA in vitro

BTV core particles were incubated at 40 μg ml^-1^ at 30°C for 5 to 6 h in core transcription buffer (100 mM Tris HCl pH8.0, 4 mM ATP, 2 mM GTP, 2 mM CTP, 2 mM UTP, 500 μM *S*-adenosylmethionine, 6 mM DTT, 9 mM MgCl_2_, 0.5U μl^-1^ RNAsin Plus [Promega]). BTV core-derived mRNAs were purified as previously described [35], followed by an additional precipitation in isopropanol and resuspension in DEPC-treated water.

### Transfection of BSR cells with BTV protein expression plasmids

70-90% confluent BSR monolayers in 96 well plates were transfected with plasmid DNA using Lipofectamine 2000™ reagent (Invitrogen), according to the manufacturer’s instructions. Incubation of transfected cells was continued at 35°C in 5% CO_2_.

### Transfection of BSR cells with reporter mRNA transcripts

Growth medium was replaced with 100 μl fresh growth medium 18 h after transfection with BTV protein expression plasmids. Reporter transcripts were diluted in OptiMEM-1, 0.1U μl^-1^ RNAsin Plus (Promega), before mixing with Lipofectamine™ 2000 reagent. Monolayers were transfected with 25 ng of the reporter transcript unless otherwise indicated, and incubated at 35°C in 5% CO_2_ for 20 h.

### Quantification of reporter expression

GFP expression in transfected BSR cells was visualised using a Nikon Eclipse TS100 microscope and recorded using a Nikon Coolpix 995 digital camera, with the number of fluorescing cells per visual field recorded. When transfection included a complete infectious set of BTV genome segments then fixation in 4% paraformaldehyde in PBS for 10 minutes was performed prior to recording fluorescence.

The expression of Renilla luciferase in transfected or infected BSR cells was quantified with a Turner Biosystems Glomax luminometer using the Dual Luciferase Reporter Assay System (Promega) or the Renilla Luciferase Assay System (Promega), according to the manufacturer’s instructions. The mean and standard deviation were calculated for triplicate monolayers transfected or infected in 96 well plates.

### Transfection of BSR cells to generate BTV-1 S10Rluc reporter virus

The S10-Rluc reporter RNA was rescued into the BTV-1 genetic background as previously described, using the BSR NS3 cell line to complement deletion of the NS3 ORF [24,26].

### Primers

Mutagenic primers used to generate variants of pS10-GFP were the following:

**Mutants 1 and 5** (Introduce a termination codon at the C terminus of the GFP ORF and a −1 frame shift of NS3 relative to GFP); S10GFP_mut1F (5^′^GGCATGGACGAGCTGTACAAGTAAGTGGAAGTTCAAGATCTATCCCG3^′^) and S10GFP_mut1R (5^′^CGGGATAGATCTTGAACTTCCACTTACTTGTACAGCTCGTCCATGCC3^′^).

**Mutants 2, 4 and 5** (NS3 initiation codon changed to AAG); S10GFP_mut2F (5^′^CTATAGTTAAAAAGTGTCGCTGCCAAGCTATCCGGGCTGATCC3^′^) and S10GFP_mut2R (5^′^GGATCAGCCCGGATAGCTTGGCAGCGACACTTTTTAACTATAG3^′^).

**Mutant 4 and 5** (NS3 2^nd^ ATG codon changed to ACG); S10GFP_mut3F (5^′^CCAAAGGTTCGAAGAAGAAAAGACGAAACACAATCAAGATAGAGTTGAAG3^′^) and S10GFP_mut3R (5^′^CTTCAACTCTATCTTGATTGTGTTTCGTCTTTTCTTCTTCGAACCTTTGG3^′^).

**Mutants 4 and 5** (Reading frame altered so that GFP is in the +1 frame compared to NS3 N terminus); S10GFP_mut4F (5^′^CGAGTGCACCGACGCGTATCCAGTGGTGGAGGTATGGTG3^′^) and S10GFP_mut4R (5^′^CACCATACCTCCACCACTGGATACGCGTCGGTGCACTCG3^′^).Mutagenic bases are underlined.

## Competing interests

The authors declare that they have no competing interests.

## Authors’ contributions

MB conceived and designed the experiments. MB and CC performed the experiments. MB and PR analyzed the data. MB and PR wrote the manuscript. All authors read and approved the final manuscript.
